# Evaluation of malaria preventive measures among adult patients attending the Bamendjou and Foumbot district hospitals of the West Region of Cameroon

**DOI:** 10.1186/s12936-021-03592-7

**Published:** 2021-01-22

**Authors:** Nfor Omarine Nlinwe, Yengong Clinton Singong, Tenkam Makamdoum Ruth Florentine

**Affiliations:** Department of Medical Laboratory Science, The University of Bamenda, Faculty of Health Sciences, Bambili, P.O Box 39, Bamenda, North West Region Cameroon

**Keywords:** Malaria, Preventive measures, Long-lasting insecticidal nets, Insecticidal sprays, Risk

## Abstract

**Background:**

Although a significant decrease in entomological and epidemiological indicators was reported in Cameroon since the introduction of insecticide-treated bed nets, malaria prevalence remains high also in some parts of the West Region of Cameroon. This study was designed to evaluate malaria preventive measures among patients attending the Bamendjou and Foumbot District hospitals of the West Region of Cameroon.

**Methods:**

This was a cross-sectional study carried out within a period of 3 months, from January to March 2020. Data was obtained using a structured questionnaire and laboratory analysis. The *CareStart*™ Pf Malaria HRP2 qualitative rapid diagnostic test was used for malaria diagnosis. The questionnaire was designed to collect information on respondent’s socio-demographic characteristics, and the use of malaria preventive measures. Data were analysed using descriptive statistics, regression analysis, and Chi-square (and Fisher’s exact) test.

**Results:**

A total of 170 study participants were recruited in Foumbot and 197 in Bamendjou. Malaria was significantly (P < 0.0001) more prevalent in Foumbot (47.06%) than in Bamendjou (19.8%). In Foumbot, non-use of insect repellent spray (P = 0.0214), insect repellent body cream (P = 0.0009), mosquito spray (P = 0.0001) and not draining stagnant water (P = 0.0004) predisposed to higher risk of malaria. In Bamendjou, non-use of insect repellent spray (P = 0.0012), long-lasting insecticidal bed nets (P = 0.0001), window and door nets (P = 0.0286), predisposed to a higher risk of malaria.

**Conclusions:**

Malaria prevalence was high among the study participants especially in Foumbot. An adequate follow-up to ensure effective execution of the recently launched third phase of LLINs distribution campaign in Cameroon is recommended. Additionally, integrated vector management is required to ensure effective control of malaria transmission in Foumbot and Bamendjou.

## Background

The estimated yearly suspected malaria cases in Cameroon, is 3.3–3.7 million in health services [1]. In Cameroon the main method of malaria prevention is the use of different types (e.g. PermaNet, Olyset, Interceptor) of long-lasting insecticidal nets (LLINs) [2]. There have been three free distribution of ITNs/LLINs campaigns; in 2004–2005 (2 million ITNs), 2011 (8 million LLINs), and in 2015 (over 12 million LLINs) [1, 3]. National coverage is anticipated with the third mass distribution campaign of LLINs launched in February 2019 [4]. A significant decrease in entomological and epidemiological indicators was reported in Cameroon since the introduction of ITNs/LLINs [5, 6]. In contrast, in the west region, a high prevalence (53.4%) of malaria was recently reported among pregnant women in Foumban, a neighbouring town to Foumbot [7]. It was reported that increased access to impregnated mosquito bed nets is needed to reduce the risk of malaria infection [7]. With an increase in coverage rates and correct usage, LLINs could greatly assist in malaria elimination in Cameroon [5].

Despite nation-wide sensitization campaigns [8], the disparity between possession and actual usage has affected the performance of LLINs at the different epidemiological settings in Cameroon [8–13]. In Cameroon, door-to-door visits of households to physically assist with hang-up of LLINs and behaviour change communication (BCC) campaign scaled up the use of bed nets from 75 to 92% after the campaign [4]. During the door-to-door mass distribution of LLNs in Zambia, the practice of net hanging and face-to-face health education on adequate use to prevent wear and tear of LLNs, increased its usage and coverage rates [14]. Therefore, the effectiveness of LLINs could be well-maintained by evaluating their quality, sustainable usage, insecticidal persistence, and efficacy with changing seasons. Indoor residual spraying and larviciding can effectively complement the existing malaria transmission control strategies [15]. Also, the effect of hygiene and sanitation on the reduction of permanent mosquito breeding sites cannot be overemphasized. Because the mosquito species in the study area, *Anopheles gambiae*, *Anopheles coluzzii* and *Anopheles. funestus,* are among the malaria vectors in sub-Saharan Africa (SSA) whose larvae breed near human habitats [16], environmental hygiene is a possible control strategy. Routine epidemiological investigative activities are requested to monitor changes in malaria occurrence, mosquito biting, entomological inoculation rate, and insecticide resistance [5]. In Cameroon, the following anopheline species transmit malaria parasites: *An. gambiae, An. coluzzii*, *An. funestus, Anopheles arabiensis*, *Anopheles moucheti* and *Anopheles nili* [5, 15]. The performance of LLINs has been threatened by an increase in carbamate, pyrethroid, and DDT resistance in the main malaria vectors in the West region of Cameroon [5], vectors which are among the most effective vectors in SSA [17]. Although they are principally endophagic and endophilic [18], they have been shown to exhibit some degree of outdoor biting and resting [17].

Malaria research uptake on preventive measures is fundamental in a socio-variable community like Cameroon [19]. The investigation of combined preventive measures could provide valuable insights helpful in the update of control strategies. Moreover, due to an increase in insecticide resistance, the use of combined interventions is recommended in malaria hyperendemic areas [20]. Even in areas with seasonal malaria parasite transmission, combining insecticide resistance sprays and LLINs is helpful [20]. In Cameroon, challenges associated with malaria control strategies could be effectively handled if considered according to defined local epidemiological settings. Varied malaria endemicity has been reported in different localities of the West region of Cameroon. For example, Bamendjou is hypoendemic for malaria and has seasonal malaria parasite transmission [5]. Whereas malaria transmission in Foumbot is stable with most infections being asymptomatic [7]. Therefore, this study was designed to evaluate malaria preventive measures among patients attending the Bamendjou and Foumbot district hospitals of the West Region of Cameroon.

## Methods

### Study area

The West region of Cameroon has a rainfall lasting about 8 months and is situated in the highland areas. Generally, this region has a temperate climate with dominant grassland vegetation and average annual rainfall estimated at 1800 mm/year lasting for about 8 months. The West region has an estimated population of 1.9 million and covers an area of 13,892 km^2^. Before the free LLINs campaigns, malaria prevalence in this region was estimated to be 25% in children [21, 22]. Meanwhile, after free LLINs campaigns, malaria prevalence was estimated to vary from 9.3 to 22.4% [23, 24]. In 2010, the entomological inoculation rate in this region was shown to fluctuate from 62.8 to 90.5 infective bites/person/year [25]. Whereas in 2018, the entomological inoculation rate in the West region was 2.24 infective bites/person/month [25, 26].

Foumbot, located at Latitude 5° 30′ 00ʺ N, Longitude 10° 37′ 59ʺ E, and average altitude 1071 m has an equatorial climate with two climatic seasons and four ecologically dry months. Foumbot covers an area of 579 km^2^ with an estimated population of 77,130 inhabitants and located 25 km from the West Regional headquarter, Bafoussam. The main economic activity of more than 84% of the inhabitants of Foumbot remains agriculture. The rest of the inhabitants practice agriculture as a secondary activity [27]. Bamendjou, located at Latitude 5° 23′ 55.99ʺ N, Longitude 10° 18′ 60.00ʺ E and average altitude 1595 m has an equatorial climate and an average rainfall of 1500 mm, usually lasting 9 months (March to November). Bamendjou covers an area of 197 km^2^ with an estimated population of 34,269 inhabitants and located 15 km from the West Regional headquarter, Bafoussam. The main economic activity of the inhabitants of Bamendjou is agriculture and animal husbandry. The Bamendjou council signed a contract with a hygiene and sanitation company since 2018 for the cleaning and maintenance of the city centre and also received a national award for its role in promoting good governance [28].

### Study design/study participants

This is a cross-sectional study carried out for three months, starting from January to March 2020. The inclusion criteria for the study were all adult (≥ 18 years) febrile patients attending the Bamendjou and Foumbot district hospitals within the study period and who were sent to the laboratory for a malaria test. Patients who gave their consent by signing the informed consent were consecutively enrolled in the study within the study period. The limitations of sampling patients who attend hospital are that patients who do not attend hospital will not be sampled, limiting the generalization of the results to just the population of patients who attend the hospital.

The sample size calculation was done using the following formula:

$$ {\text{N}} = {\text{z}}^{{2}} {\text{pq}}/{\text{d}}^{{2}} \left[ {{29}} \right] $$

where z^2^ = (1.96)^2^; p (previous malaria prevalence) = 29% (0.29) (9). q = (1–0.224); d^2^ = (0.05)^2^; N = minimum sample size (316).

### Ethical considerations

The ethical clearance for this study was gotten from the Ethical Review Committee of the University of Bamenda. The ethical clearance numbers are 2020/0148H/UBa/IRB and 2020/0142H/UBa/IRB for data collection in Foumbot and Bamendjou, respectively. Signed informed consent was acquired from those who accepted to be enrolled in the study.

### Data collection

Data was obtained using a structured questionnaire and laboratory analysis. The *CareStart*™ Pf Malaria HRP2 qualitative rapid diagnostic test was used for malaria diagnosis, using about 5 µL of capillary blood collected by a finger prick [30]. The questionnaire was designed to collect information on respondent’s socio-demographic characteristics, and the use of malaria preventive measures. The socio-demographic characteristics were gender, age, marital status, educational level, religion, internal displacement status, monthly income, and occupation. The preventive measures under consideration were: use of LLINs, use of window and door nets, use of insect repellent spray, draining stagnant water, killing a mosquito with a broom, use of mosquito coil, use of insect repellent body cream, use of mosquito candles and use of mosquito spray. Non-use of malaria preventive measures by study participants were considered exposed to malaria.

### Data analysis

Baseline characteristics of malaria preventive measures and socio-demographic factors of patients with or without malaria were determined using excel. The baseline characteristics include sums and mean percentages. Amongst patients in Foumbot and Bamendjou communities, the difference in malaria occurrence and socio-demographic characteristics were determined using an independent t-test. Regression analysis was used to evaluate the association between malaria incidence and sociodemographic factors. Malaria occurrence was considered the dependent variable and socio-demographic factors, the independent variables. A fourfold (2 × 2) contingency table displaying the frequency distribution for each malaria preventive measure was entered into Graph Pad Prism version 8.2.1. In each of the four cells, the contingency table had frequencies for use and non-use of preventive measures by both the negative and positive malaria cases. Chi-square (and Fisher’s exact) test was used to determine the relative risk, attributable risk, odds ratio, and likelihood ratio of malaria occurrence in malaria exposed patients. The sensitivity and specificity for the prediction of the risk of malaria in exposed patients were also determined by Chi-square (and Fisher’s exact). Results were determined at a 95% confidence level. Graph Pad Prism version 8.2.1 was used for all statistical analyses.

## Results

A total of 367 patients were recruited for the study with a total malaria prevalence of 32.43% (119/367). Malaria was significantly (P < 0.0001) more prevalent among the study participants attending the Foumbot district hospital (47.06%) than those attending the Bamendjou (19.8%) district hospital. The female to male ratios were 1.33:1 and 4.27:1 in Foumbot and Bamendjou respectively. There were significant differences in the distribution of gender, age, marital status, educational level, religion, internal displacement status, and occupation, among the study participants in the Foumbot and Bamendjou district hospitals (Table 1).

**Table 1:** Socio-demographic data of study participants in Foumbot and Bamendjou district hospitals

	Study area	Foumbot			Bamendjou			P value
	Diagnostic test	RDT Pos (%)	RDT Neg (%)	Total (%)	RDT Pos (%)	RDT Neg (%)	Total (%)	
	Number examined	80 (47.06)	90 (52.94)	170	39 (19.8)	158 (80.20)	197	< 0.0001
Sex	Females	55 (68.75)	42 (46.67)	97 (57.06)	30 (76.92)	128 (81.01)	158 (80.2)	< 0.0001
	Males	25 (31.25)	48 (53.33)	73 (42.94)	9 (23.08)	30 (18.99)	39 (19.8)	
Age (years)	18–30	30 (37.5)	30 (33 .33)	60 (35.29)	11 (28.21)	29 (18.35)	40 (20.3)	
	31–40	25 (31.25)	30 (33.33)	55 (32.35)	10 (25.64)	57 (36.08)	67 (34.01)	0.0008
	41–50	15 (18.75)	20 (22.22)	35 (20.59)	9 (23.08)	44 (27.85)	53 (26.9)	
	> 50	10 (12.5)	10 (11.11)	20 (11.76)	9 (23.08)	28 (17.72)	37 (18.78)	
Marital status	Single	50 (62.5)	50 (55.56)	100 (58.82)	18 (46.15)	60 (37.97)	78 (39.59)	
	Married	30 (37.5)	35 (38.89)	65 (38.24)	15 (38.46)	72 (45.57)	87 (44.16)	0.0019
	Widow/widower	0	0	0	6 (15.38)	24 (15.19)	30 (15.23)	
	Divorced	0	5 (5.56)	5 (2.94)	0	2 (1.27)	2 (1.02)	
Educational level	No formal education	0	5 (5.56)	5 (2.94)	4 (10.26)	22 (13.92)	26 (13.2)	
	Primary	10 (12.5)	30 (33.33)	40 (23.53)	10 (25.64)	42 (26.58)	52 (26.4)	< 0.0001
	Secondary level	55 (68.75)	30 (33.33)	85 (50%)	19 (48.72)	78 (49.37)	97 (49.24)	
	Higher education	15 (18.75)	25 (27.78)	40 (23.53)	6 (15.38)	16 (10.13)	22 (11.17)	
Religion	Christian	50 (62.5)	35 (38.89)	85 (50)	36 (92.31)	141 (89.24)	177 (89.85)	
	Moslem	25 (31.25)	55 (61.11)	80 (47.06)	0	3 (1.2)	3 (1.52)	< 0.0001
	Others	5 (6.25)	0	5 (2.94)	3 (7.69)	14 (8.86)	17 (8.63)	
Displacement status	An IDP?	20 (25.0)	15 (16.67)	35 (20.59)	1 (2.56)	14 (8.86)	15 (7.61)	0.0003
	Not an IDP	60 (75.0)	75 (83.33)	135 (79.41)	38 (97.44)	144 (91.14)	182 (92.39)	
Monthly income (frs)	Low (< 30,000)	40 (50.0)	50 (55.56)	90 (52.94)	28 (71.79)	123 (77.85)	151 (76.65)	
	Medium 30,000–250,000)	35 (43.75)	40 (44.44)	75 (44.12)	9 (23.08)	33 (20.89)	42 (21.32)	0.0527
	High > 250,000	5 (6.25)	0	5 (2.94)	2 (5.13)	2 (1.27)	4 (2.03)	
Occupation	Civil servants	5 (6.25)	0	5 (2.94)	8 (20.51)	17 (10.76)	25 (12.69)	
	Business	5 (6.25)	5 (5.56)	10 (5.88)	2 (5.13)	22 (13.92)	24 (12.18)	0.0035
	Farmer	30 (37.5)	34 (37.78)	64 (37.65)	11 (28.21)	49 (31.01)	60 (30.46)	
	Others	40 (50.0)	51 (56.67)	91 (53.53)	18 (46.15)	70 (44.30)	88 (44.67)	

There was no significant association between sociodemographic factors and malaria incidence among the study participants attending the Bamendjou district hospital. However among those attending the Foumbot district hospital, being a female (P = 0.0001), Christianity (P < 0.0001), increased educational level (P < 0.04) and decreased monthly income (P < 0.0001) were significantly associated with the likelihood of malaria (Table 2).

**Table 2:** Summary of regression analysis of socio-demographic data

Variable	Foumbot		Bamendjou	
	|t|	P value	|t|	P value
Intercept	3.529	0.0005*	0.9429	0.3469
Sex	3.93	0.0001***	0.3365	0.7369
Age	0.9931	0.3222	0.1962	0.8447
Marital status	1.281	0.2019	0.5281	0.598
Religion	6.075	< 0.0001****	0.3396	0.7345
Educational level	2.071	0.04*	0.1516	0.8796
Occupation	0.06659	0.947	0.01536	0.9878
Are you an IDP?	0.5037	0.6152	1.182	0.2387
Monthly income	3.133	0.0021**	0.9842	0.3263

*Significant P-values

Among the study participants attending the Foumbot district hospital, the most used preventive measures were LLINs (79.41%) and window and door nets (70.59%). Meanwhile, the least was to kill mosquitoes with a broom (23.53%), mosquito candles (26.47%), and mosquito sprays (26.47%). Among the study participants attending the Bamendjou district hospital, the most used preventive measures were window and door nets (87.82%) and the least was insect repellent body cream (8.63%), mosquito candles (13.2%), and mosquito sprays (15.23%) (Table 3).

**Table 3:** Preventive measures used by the study participants for malaria control

	Foumbot community			Bamendjou community		
	RDT Pos (%), N = 80 (47.06)	RDT Neg (%), N = 90 (52.94)	Total (%), N = 170	RDT Pos (%), N = 39 (19.8)	RDT Neg (%), N = 158 (80.20)	Total (%), N = 197
Use of LLINs	60 (75)	75 (83.33)	135 (79.41)	30 (76.92)	45 (91.77)	75 (38.07)
Use of window and door nets	55 (68.75)	65 (72.22)	120 (70.59)	30 (76.92)	143 (90.51)	173 (87.82)
Using insect repellent spray	30 (37.5)	50 (55.56)	80 (47.06)	10 (25.64)	87 (55.06)	97 (49.24)
Draining stagnant water	40 (50)	69 (76.67)	109 (64.12)	20 (51.28)	90 (56.96)	110 (55.84)
Killing mosquito with a broom	20 (25)	20 (22.22)	40 (23.53)	18 (46.15)	50 (31.65)	68 (34.52)
Using mosquito coil	25 (31.25)	35 (38.89)	60 (35.29)	10 (25.64)	28 (17.72)	38 (19.29)
Insect repellent body cream	15 (18.75)	39 (43.33)	54 (31.76)	3 (7.69)	14 (8.86)	17 (8.63)
Use of mosquito candle	25 (31.25)	20 (22.22)	45 (26.47)	3 (7.69)	23 (14.56)	26 (13.2)
Use of Mosquito sprays	10 (12.5)	35 (38.89)	45 (26.47)	5 (12.82)	25 (15.82)	30 (15.23)

Among the study participants in the Foumbot district hospital, non-use of insect repellent spray, insect repellent body cream, mosquito spray, and not draining stagnant water, were all significantly associated with increased relative risk, attributable risk, odds ratio, and likelihood ratio. Non-use of these preventive measures was equally significantly associated with good sensitivity and specificity for the prediction of risk of malaria, but for non-use of mosquito spray, with poor specificity (38.89%) (Table 4).

**Table 4:** Risk of malaria occurrence among exposed study participants in Foumbot district hospital

Variable	Relative risk (95% CI)	Attributable risk (95% CI)	Odds ratio (95% CI)	Sensitivity (95% CI)	Specificity (95% CI)	LR	P-value
Non-use of LLINs	1.29 0.88 to 1.76	0.13 − 0.07 to 0.31	1.67 0.81 to 3.65	25 16.81 to 35.48	83.33 74.31 to 89.63	1.5	0.1898
Non-use of window and door nets	1.09 0.76 to 1.5	0.04 − 0.13 to 0.21	1.82 0.62 to 2.22	31.25 22.15 to 42.07	72.22 62.20 to 80.42	1.23	0.7362
Non-use of insect repellent spray	1.48 1.07 to 2.1	0.18 0.02 to 0.33	2.08 1.11 to 3.82	62.5 51.55 to 72.31	55.56 45.27 to 65.38	1.41	0.0214*
Not draining stagnant water	1.79 1.31 to 2.43	0.29 0.12 to 0.43	3.29 1.72 to 6.29	50 39.3 to 60.7	76.67 66.95 to 84.2	2.14	0.0004***
Not killing mosquito with a broom	0.92 0.66 to 1.37	0.04 − 0.14 to 0.22	0.86 0.41 to 1.79	75 64.52 to 83.19	22.22 14.87 to 31.85	0.96	0.7191
Non-use of mosquito coil	1.2 0.86 to 1.74	0.08 − 0.08 to 0.23	1.4 0.75 to 2.68	68.75 57.93 to 77.85	38.89 29.47 to 49.22	1.13	0.3366
Non-use of insect repellent body cream	2.02 1.32 to 3.26	0.29 0.11 to 0.42	3.31 1.63 to 6.68	81.25 71.34 to 88.29	43.33 33.58 to 53.64	1.43	0.0009***
Non-use of mosquito candle	0.79 0.58 to 1.12	0.12 − 0.05 to 0.3	0.63 0.31 to 1.23	68.75 57.93 to 77.85	22.22 14.87 to 31.85	0.88	0.2234
Non-use of mosquito spray	2.52 1.5 to 4.55	0.34 0.16 to 0.47	4.46 2.03 to 9.42	87.5 78.5 to 93.07	38.89 29.47 to 49.22	1.43	0.0001***

*Significant P-values

Among the study participants in the Bamendjou district hospital non-use of insect repellent spray and window, and door nets, were significantly associated with increased relative risk, attributable risk, odds ratio and likelihood ratio. Non-use of window and door nets was significantly associated with poor sensitivity and very good specificity, for the prediction of risk of malaria. Non-use of insect repellent spray was significantly associated with good sensitivity and average specificity, for the prediction of risk of malaria. However, the non-use of LLINs was rather significantly associated with decreased relative risk, attributable risk, odds ratio, and likelihood ratio, but also associated with poor sensitivity and specificity for the prediction of risk of malaria infection (Table 5).

**Table 5:** Risk of malaria occurrence among exposed study participants in the Bamendjou district hospital

Variable	Relative risk (95% CI)	Attributable risk (95% CI)	Odds ratio (95% CI)	Sensitivity (95% CI)	Specificity (95% CI)	LR	P-value
Non-use of LLINs	0.18 0.093 to 0.36	0.33 0.2 to 0.45	0.12 0.05 to 0.28	23.08 12.65 to 38.34	24.48 22.02 to 35.96	0.32	< 0.0001****
Non-use of window and door nets	2.16 1.13 to 3.77	0.2 0.01 to 0.43	2.86 1.2 to 7.36	23.08 12.65 to 38.34	90.51 84.93 to 94.16	2.43	0.0286*
Non-use of insect repellent spray	2.81 1.48 to 5.44	0.19 0.07 to 0.3	3.55 1.62 to 7.48	74.36 58.92 to 85.43	55.06 47.28 to 62.61	1.66	0.0012**
Not draining stagnant water	1.2 0.69 to 2.09	0.04 − 0.08 to 0.16	1.26 0.61 to 2.58	48.72 33.87 to 63.80	56.96 49.17 to 64.43	1.13	0.5904
Not killing mosquito with a broom	0.57 0.33 to 0.1	0.11 − 0.02 to 0.23	0.49 0.24 to 0.98	53.85 38.57 to 68.43	29.76 23.36 to 37.07	0.77	0.0592
Non-use of mosquito coil	0.69 0.39 to 1.32	0.08 − 0.1 to 0.22	0.62 0.28 to 1.41	74.36 58.92 to 85.43	17.72 12.56 to 24.42	0.9	0.2639
Non-use of insect repellent body cream	1.13 0.46 to 3.34	0.02 − 0.25 to 0.17	1.17 0.36 to 3.98	92.31 79.68 to 97.35	8.86 5.35 to 14.32	1.01	> 0.9999
Non-use of mosquito candle	1.83 0.69 to 5.43	0.1 − 0.11 to 0.21	2.04 0.63 to 6.74	92.31 79.68 to 97.35	14.56 9.9 to 20.9	1.08	0.3048
Non-use of mosquito spray	1.22 0.56 to 2.91	0.04 − 0.16 to 0.16	1.28 0.47 to 3.25	87.18 73.29 to 94.40	15.82 10.95 to 22.31	1.04	0.8051

*Significant P-values

## Discussion

The study participants attending the Foumbot and Bamendjou district hospitals differ in their socio-demographic characteristics, except monthly income. Malaria endemicity in Foumbot and Bamendjou also differ, as earlier reported [5, 7]. Thus, adequate attention to socio-demographic characteristics is important in malaria control efforts [31]. Among study participants attending the Bamendjou district hospital, the female to male ratio was 4.05:1. The malaria positive female to male ratio was 3.33:1 and malaria negative female to male ratio was 4:1. Among study participants attending the Foumbot district hospital, the female to male ratio was 1.33:1. The malaria positive female to male ratio was 2.2:1 and the malaria negative female to male ratio was 0.88:1. Therefore, in addition to females constituting the majority of the study participants, they were also more infected. In line with findings from other studies, higher malaria prevalence among females can be associated with exposure patterns, influenced by socio-economic roles [32, 33]. An earlier study suggested that poverty-related issues affected female adoption of malaria control methods [34]. However, the proportion of infected males increased among the study participants in the Bamendjou district hospital, which had lower malaria prevalence. This may be due to the perceived reduced need for additional malaria control efforts.

The 31–40 years age group was most represented among the study participants attending the Bamendjou district hospital while the 18–30 years age group were most represented among those attending the Foumbot district hospital. The ˃ 50 years age group was the least represented in both communities. With a higher malaria prevalence among the participants in Foumbot district hospital, the young adults (18–30 years) age group was generally more at risk of malaria than the other age groups. Although the middle-aged adult (31–40 years) group had the highest malaria prevalence among those attending the Bamendjou district hospital, this community generally had low malaria prevalence. Similar to findings from another study in the North West region of Cameroon [35], the young adult age group is more at risk of malaria. Although children < 5 years and pregnant women are naturally more predisposed to malaria [36–39], differences in exposure patterns may also increase the risk of malaria among young adults. Compared to other age groups, young adults are more involved in outdoor activities like farming and could be casual towards malaria preventive measures.

There were more Muslims among the study participants in the Foumbot district hospital which had more malaria positive cases. Other studies reported a strong correlation between religion and health-seeking behaviour towards malaria control and prevention [40–42]. Most of the patients in both communities had a secondary school level of education. In line with findings from other studies [43–45], education can moderate religious perceptions towards malaria prevention and control. With the current socio-political crisis in the North West and southwest regions of Cameroon, the West region has experienced a huge influx of internally displaced persons from the crisis plagued regions. The living conditions of the displaced persons are usually of lower quality, predisposing them to malaria and also probably to new strains of malaria parasites [34]. In addition to malaria prevalence being higher among the participants attending the Foumbot district hospital, there were more internally displaced patients in the Foumbot district hospital. Contrary to findings from Bamendjou, gender, religion, educational level, and financial status were significantly associated with malaria among those attending the Foumbot district hospital, with Foumbot also having a history of steady malaria transmission [7]. In line with findings from malaria-risk areas, religion, education, and income were found to impact the use of ITN [42], which directly influences malaria transmission.

Although LLINs was the most used malaria preventive measure among the study participants attending the Foumbot district hospital, non-use of it was not significantly associated with the risk of malaria. But among participants attending the Bamendjou district hospital, non-use of LLINs was rather significantly associated with a lower risk of malaria exposure. However, the sensitivity (23.08%) and specificity (24.48%) of LLINs usage to predict the risk of malaria were low. Generally, LLINs usage was a poor indicator for the prediction of the risk of malaria. This could be explained by low and inconsistent usage rate, not sleeping under the nets at the time of biting, not using at night due to nocturnal activities, poor maintenance of LLINs, in addition to biological and behavioral changes in the mosquito vector. A recent study in Foumban, which is located 45.2 km from Foumbot, revealed low usage of LLINs and high malaria prevalence. Malaria prevalence among pregnant women was 53.4% and only 49.3% of the study participants made use of bed nets [7]. Reduced chances of malaria infection were found among children who slept under intact nets, suggesting the importance of repair and care of ITNs by owners [46]. Several other studies have emphasized the importance of correct usage of insecticide pre-treated bed nets [5, 7, 14, 47]. Insecticide resistance also seriously threatens the effectiveness of LLINs as a malaria control tool [48]. There was scale-up in the effective use of LLINs in Baré a rural part of Cameroon, following door-to-door hang-up and behaviour change communication (BCC) campaign, after the third mass distribution campaign launched in February 2019 [4]. Therefore, with the extension of such door-to-door hang-up and (BCC campaign to other rural areas like Foumbot and Bamendjou, LLINs usage could yield better results. The current study reveals that approximately 1 year after the launching of the third LLINs campaign in Cameroon, malaria prevalence remains high especially among the patients who attended the Foumbot district hospital (47.06%).

Non-use of window and door nets was also not significantly associated with the risk of malaria among the study participants attending the Foumbot district hospital. Among the study participants attending the Bamendjou district hospital, non-use of window and door nets was significantly (P = 0.0286) associated with a higher odds of malaria. From the relative risk (2.16), the non-use of window and door nets was associated with more than 100% higher risk of malaria. This is supported by the positive attributable risk (0.2). The odds ratio of 2.86 also indicates a greater odds of malaria occurring in those who did not use window and door nets. Although the sensitivity of window and door net usage to predict the risk of malaria was low (23.08%), the specificity was high (90.51%). Therefore, it is only 23.08% likely that those who did not use window and door nets will test malaria positive. However, it is 90.51% likely that those who use window and door nets will test malaria negative. This probably explains why malaria prevalence was lower among the study participants in the Bamendjou district hospital. Furthermore, window and door nets that protects the home (accommodation area) from mosquitoes were found to be one of the effective measures against malaria [47]. Window and door nets were also considered suitable alternatives for LLINs [49]. In the current study, it is up to 90.51% likely that those who used window and door nets will test malaria negative, even though the nets were neither pre-impregnated nor sprayed. Similarly, LLINs with or without insecticidal residual spray prevented more than 99% of indoor mosquito bites [50, 51]. Therefore, the augmentation of the door and window nets usage by pre-impregnating or spraying with insecticidal may possibly improve malaria control efforts especially in low malaria transmission areas like Bamendjou.

In Foumbot, non-use of insect repellent spray and mosquito spray was significantly associated with a higher odds of testing malaria positive. The sensitivity of insect repellent spray (62.5%) and mosquito spray (87.5%) used to predict the risk of malaria, were good. However, the specificity for insect repellent spray (55.5%) and mosquito spray (38.89%) was lower. The odds of malaria occurrence in those who did not use insect repellent spray (RR: 1.48, AR: 0.18, OR: 2.08 and LR: 1.41) and mosquito spray (RR: 2.52, AR: 0.34, OR: 4.46 and LR: 1.43) were high. From their relative risks, non-use of insect repellent spray and mosquito spray was associated with 48% and ˃ 100% higher risk of malaria. This is strongly supported by their positive attributable risks. The odds ratio of 2.08 and 4.46 for non-use of insect repellent spray and mosquito spray further indicates a greater probability of malaria occurrence in the exposed individuals. The likelihood ratio of 1.41 and 1.43 for the non-use of both sprays confirms that non-use of insect repellent and mosquito sprays were associated with a higher risk of malaria in Foumbot. In Bamendjou, non-use of insect repellent spray was associated with higher risk of malaria. However, non-use of mosquito spray was not. The relative risk (2.81) for non-use of insect repellent spray indicates more than 100% risk of malaria. The odds ratio (3.55) and likelihood ratio (1.66) further indicates greater odds and association of non-use of insect repellent sprays with a higher risk of malaria in Bamendjou.

In Bamendjou, non-use of insect repellent body cream was not significantly associated with odds of malaria occurrence. However, in Foumbot, the non-use of insect repellent body cream was significantly (P = 0.0009) associated with the risk of malaria. The relative risk of 2.02 means the non-use of insect repellent body cream was associated with more than 100% higher risk of malaria. In addition to a positive attributable risk of 0.29, the odds ratio of 3.31 indicates a greater odds of malaria occurring in the exposed individuals. Furthermore, the likelihood ratio of 1.43 confirms an association between non-use of insect repellent body cream and malaria, in Foumbot.

In Foumbot which had higher malaria prevalence, non-use of insect repellent spray, cream, and mosquito spray predisposed to a higher risk of malaria. The non-use of LLINs, window and door nets, was not associated with the risk of malaria. Therefore, outdoor malaria transmission could be higher in Foumbot since malaria vectors with exophilic host-seeking and resting behaviour bites more outdoor [18, 52]. In Bamendjou, with lower malaria prevalence, non-use of insect repellent spray, LLINs, window, and door nets all predisposed to a higher risk of malaria. However, non-use of insect repellent cream and mosquito spray did not predispose to the risk of malaria. Indoor malaria transmission may be higher in Bamendjou since the use of window and door nets protected against malaria [53]. Although increasing intensities of insecticide resistance [15, 54–57] and outdoor transmission threaten the effectiveness of indoor residual spray [58], different methods of repellent deliveries (as sprays, body creams, and on bed nets) are essential [59]. Generally, the active ingredients in insect repellent sprays include picaridin, botanicals, citronella and *N*,*N*-diethyl-3-methylbenzamide (DEET). DEET, picaridin, MGK-326, MGK-264, IR3535, oil of citronella, and oil of lemon eucalyptus has been approved for skin topical application [60]. The effectiveness of each delivery may be affected by behavioural changes in both the human and vector hosts [58, 61]. These changes also include insecticide resistance patterns. In Cameroon, insecticide resistance was highly prevalent in both *An. gambiae* sensu lato (s.l.) and *An. funestus*. DDT, permethrin, deltamethrin, and bendiocarb seemed to be the most affected compounds by resistance [15]. In Foumbot, *An. gambiae* s.l. was shown to be resistant to DDT, permethrin, deltamethrin, lambda-cyhalothrin, bendiocarb, and malathion [62, 63].

In another study, although picaridin repellent reduced 97% of mosquito bites, daily use was low and the effectiveness of malaria preventive measures was found to be mainly influenced by human behavior [61]. In the current study, only 8.63% and 31.76% of the study participants used insect repellent body cream in Bamendjou and Foumbot respectively. Topical repellent plus LLINs was also not found to be a suitable intervention against malaria, in an agricultural population in southern Lao PDR [64]. Although indoor residual spraying and LLINs were reported to be the most successful approaches in malaria control [65], as suggested by the Global Malaria Control Strategy, integrated vector management methods are needed for effective vector control [65].

In both Foumbot and Bamendjou, not killing a mosquito with a broom, non-use of mosquito coil, and non-use of mosquito candle were not associated with the risk of malaria. Even though not draining stagnant water was not associated with the risk of malaria in Bamendjou, it was significantly (P = 0.0004) associated with the risk of malaria in Foumbot. The odds of malaria occurrence in those who did not drain stagnant water around homes was higher (RR: 1.79, AR: 0.29, OR: 3.29 and LR: 2.14). Those who did not drain stagnant water were 79% more at risk of malaria. Furthermore, the positive attributable risk and high odds ratio indicate higher odds of malaria occurrence in those who did not drain stagnant water. The likelihood ratio of 2.14 also confirms an association between malaria and the draining of stagnant water in Foumbot. A dirty environment has been reported to increase malaria transmission [66–70]. Although Foumbot and Bamendjou are both rural areas, unlike Foumbot, Bamendjou municipality is committed to environmental sanitation. The clean environment of Bamendjou may have contributed to the low malaria prevalence. In Foumbot the sensitivity and specificity for the use of draining stagnant water to predict malaria occurrence was 50% and 76.67%, respectively. Therefore, it is 50% likely that those who did not drain stagnant water around homes will test malaria positive and 76.67% likely that those who drained stagnant water will test malaria negative. Environmental sanitation remains a main contributing factor in controlling malaria transmission, especially in rural parts of Cameroon like Foumbot.

## Conclusions

Some of the malaria preventive measures in the current study did not sufficiently protect against malaria, especially in Foumbot which recorded a higher malaria prevalence. Differences in the effectiveness of preventive measures between Foumbot and Bamendjou suggest the need for integrated vector management. To ensure effective integrated vector management, current entomological studies on malaria transmission in these study areas are necessary. This will provide adequate insight into the behavioural ecology of malaria vectors. The proper follow-up to ensure effective execution of the recently launched third phase of LLINs mass distribution campaign in Cameroon is recommended.

## Data Availability

All data generated or analysed during this study are included in this article (and its additional information files).

## Abbreviations

LLINs: Long-lasting insecticidal bed nets
ITN: Insecticide-treated bed nets
RR: Relative risk
OR: Odds ratio
AR: Attributable risk
LR: Likelihood ratio
